# The positive impact of self-compassion on disordered eating and associated risk factors

**DOI:** 10.1186/2050-2974-2-S1-O4

**Published:** 2014-11-24

**Authors:** Tiegan Holtham, Murray Dyck

**Affiliations:** 1 Griffith University, Gold Coast, Australia

## 

Self-compassion is associated with improved affect regulation, adaptive coping strategies and various measures of wellbeing. It has also been suggested as a protective factor against characteristics considered to maintain eating pathology. Specifically, cognitive theories of eating disorders suggest that dysfunctional assumptions and negative beliefs regarding shape, weight and eating lead to ego-dysfunction characteristics (perfectionistic concerns, experiential avoidance, low self-esteem) which increase eating disorder risk. The following study explored the hypothesis that self-compassion is a critical protective element in ameliorating vulnerability factors related to disordered eating behaviours. These relationships were explored in an adult community sample (N = 423). Dysfunctional assumptions and beliefs related to eating predicted disordered eating behaviours, and this relationship was mediated by low self-esteem and experiential avoidance, but not perfectionistic concerns. Self-compassion was negatively associated with disordered eating, and was found to moderate each of the relationships between disordered eating and perfectionistic concerns, experiential avoidance and low self-esteem. At high levels of self-compassion, the relationship between the risk variables and disordered eating was no longer significant. The current study supports the use of self-compassion in prevention and intervention of eating pathology.

This abstract was presented in the **Peter Beumont Young Investigator award finalist** stream of the 2014 ANZAED Conference.

